
JOURNAL INFORMATION
==============================
NLM Title Abbreviation: Med Chir Rev
NLM Journal ID: 0034617

The Medico-Chirurgical Review

Publisher: Biomedical Journal Digitization Project

ARTICLE INFORMATION
==============================
PMCID: PMC4966261
PMID: 29917631
Article version: 1
Subjects: Reviews, Medico-Chirurgical Transactions

Cases of Tumours in the Abdomen, Arising from Organic Disease of the Stomach, with Remarks

Print publication date: 1828 Oct 1
Volume: 9
Issue: 18
First page: 399
Last page: 404
1828 Sep
License: This work is free of known copyright restrictions. For more information, please see PMC Back Issue Digitization.
License URL: https://creativecommons.org/publicdomain/mark/1.0/

==============================

supply of blood, by the obliteration of proper nutrient vessels, might have been suddenly thrown into a state of partial paralysis—hence the feebleness of its efforts, which were inadequate to excite the slightest movement in the arteries."

We have now endeavoured to exhibit a full and complete delineation of this paper, occupying more than one hundred pages of the volume before us. It does credit to the zeal and careful observation of the author, and must prove useful to his brethren at large. We return him our thanks for the information which he has afforded us.

---

### XII.

*Cases of Tumours in the Abdomen, arising from Organic Disease of the Stomach; with Remarks.* By Ed. J. Seymour, M.D. &c.

[Med.-Chir Trans. Vol. XIV.]

Notwithstanding the pliancy of the abdominal parietes, and the facilities which we possess of manual examinations there, in addition to general and local symptoms, yet it is certain that medical men are far more puzzled by abdominal than thoracic diseases. We have often drawn the attention of our brethren to this subject, and have now another opportunity, in the analysis of Dr. Seymour's paper recently published.

Several cases have lately fallen under Dr. S.'s notice, in which tumours of considerable size were found to spring from the stomach, and in two of these the organ was changed in structure, without exhibiting corresponding symptoms during life. Such instances are by no means uncommon, though very inexplicable. The case of Napoleon was an example which attracted the attention of the whole world at the time. Dr. Monro states the case of a lady, who had suffered, during some time, from pain in the epigastric region, indigestion, and flatulence. On examination before death, a tumour was found in the right side of the navel, of an oval shape, and the size of an orange. It was supposed to be situated in the colon; but, on dissection, the stomach was found to have descended as low as the umbilicus, and, on opening it, there appeared a tumour adhering by its neck to the villous coat. It was of smooth surface—cold—tough—and difficult to cut through. Dr. Baillie tells us, that

"where the person is much emaciated, and the cancerous swelling is situated near the pylorus, or along a part of the great curvature of the stomach, it may be felt in the living body by a careful examination by the hand." 224.

This, as Dr. Seymour observes, merely implies that, in certain cases, such enlargements may be discovered; whereas, in Dr. S's. cases, the tumours were visible and very large.

"The essential symptoms of cancer or fungus hæmatodes of the pylorus, enumerated by authors, are pain in the region of the stomach aggravated on taking food, frequent vomiting sometimes mixed with blood, often occurring about half an hour after solids or fluids have been swallowed, sensation of weakness, occasional syncope. As the disease advances, the vomiting increases in frequency and resembles coffee in colour, and there are often accessions of hectic fever, with great emaciation.

"The disease very rarely, if ever, attacks persons under forty years of age, and it is more common in women than in men. It has been very frequently observed in persons subjected either to great fatigue of mind or anxiety. In a great majority of cases there is a remarkably exsanguine appearance in the countenance, even early in the disease.

"In the numerous cases related by M. Lieutaud, vomiting was always present, and in the greater number acute pain was experienced in the stomach. M. Vicq. d'Azyr, in his very able article Anatomie Pathologique, in speaking of this disease, says, 'It is worthy of remark, that as there is *always vomiting* when the seat of the malady occupies the pylorus, or its neighbourhood, so deglutition is impeded or altogether obstructed when the disease attacks the cardiac orifice.'

"'When the disease,' says Dr. Monro, 'is seated in the stomach, there is great pain in the organ affected, with all the usual symptoms of indigestion, very frequent nausea and vomiting, and the occasional rejection of blood by vomiting, and the patient dies completely exhausted.'" 225.

But although the above symptoms are generally present, they are not always so. Dr. Pemberton has related a case where very extensive disease existed in the stomach, and yet no symptom appeared during life to indicate such disorganization. Andral has recently related some cases of fungus hæmatodes of the pylorus, in the greater number of which there was no pain, but where the emaciation and vomiting, together with the *occasional* detection of hardness on pressure, "rendered it difficult *not* to perceive the nature of the disease."* M. Andral is, nevertheless, of opinion, that no symptoms exist, which can, in the living body, point out the diagnosis between this disease, and chronic inflammation of the stomach. We shall now proceed to the cases brought forward by Dr. Seymour.

*Case* 1. Ann Row, æt. 39, unmarried, was received into St. George's hospital on the 11th July, 1827. She had complained of pain in the abdomen for several years past, and about the preceding Christmas was affected with hæmatemesis and diarrhœa, attended with violent pain. Two months prior to her entrance into hospital, she perceived a tumour at the inferior part of the left hypochondrium, stretching to the umbilicus.

"The tumour which is hard, unequal, and very tender on pressure, occupies the whole of the epigastric and the umbilical region, extending to within an inch of the symphysis pubis and to the right iliac region; at this latter part (an inch to the right of the navel) it is more elevated, and there is a strong pulsation communicated through it. She vomits occasionally after taking food, but not always; sometimes when the stomach is empty. She describes what she vomits to be bitter and sour. Bowels very much relaxed, tongue clean and moist and of natural colour; no catamenia for three months; pulse 96, very weak; urine scanty; she is much emaciated." 227.

Leeches, saline draughts, fomentations, oxide of bismuth, &c. were employed, but on the 18th of the same month, we find a diffused swelling about the

* This is the expression of Dr. Seymour, and we leave it to the sense of practitioners, whether it is the most appropriate which could have been used. It may be a difficult thing for Dr. S. to be deceived in the detection of an *internal* "fungus hæmatodes," but then he should have some mercy on his brethren, who have very great difficulty sometimes in detecting the nature of such an affection.

right clavicle, extending to the axilla, and very tender to the touch. A purging came on—emaciation advanced—and she died on the 25th of July.

*Dissection.* "The cardiac extremity of the stomach was healthy; but on cutting the anterior surface of the pyloric portion, the coats were found considerably thickened, and on the inner surface an irregular tumour presented itself, occupying about two thirds of the circumference of the stomach, and only leaving the anterior part free. The tumour began about the situation of the pylorus, and its greatest length was about five inches extending towards the left side. It projected about an inch into the interior of the stomach, the surface being very uneven, several round masses rising upwards from the body of the tumour. The surface was for the most part of a reddish yellow colour, some parts nearly brown, and here and there complete sloughs had been formed. The surface of the duodenum and of the stomach was very vascular around the tumour.

"In the centre of the tumour an opening about an inch and a half in diameter, with sloughy circular margin, led backwards into a cavity containing about two ounces of fetid pus. The whole surface of the cavity being covered with a brown sloughy membrane like the margin of the opening, its parietes were formed by adhesions between the stomach, colon, and duodenum anteriorly, and by the spine behind. At the margin of the opening of the stomach nearest the duodenum, a sloughy tumour about the size of a small orange projected from the general mass into the abscess, and still more to the right side another larger tumour was perceptible, both from the front of the abdomen and at the bottom of the diseased mass of the intestines and stomach, having the duodenum, colon, and stomach adherent to the anterior surface. This was the only part of the whole disease which had not yet ulcerated, and it seemed to be composed of glands united together; it was soft and pulpy and of a light colour, like the usual appearance of fungus hæmatodes. The remainder where ulcerated was also soft, and resembled very much the usual surface of a tumour composed of fungus hæmatodes when it has ulcerated through the common integuments." 230.

*Case 2.* Mr. C. aged 50, who had enjoyed good health, and was temperate, but who was exposed to anxiety of mind, consulted Dr. S. in November, 1825, for a pain felt in the region of the bladder, especially after voiding urine, which contained much uric acid. The warm bath, soda, opium, relieved this complaint. In November, 1826, he complained of water-brash, unattended with pain; but his appetite was good, as was his health generally. He was ordered to take twenty minims of the liquor potassæ in lime-water twice a day, which he did not take. On the 13th March, 1827, Dr. S. accidentally saw this gentleman, and remarked that his countenance and manner betrayed great indisposition. On being stripped and examined in bed, the following phenomena presented themselves.

"About midway between the umbilicus and superior anterior spinous process of the left ilium, a tumour was observed of the size of a large orange, extremely hard, extending over about half an inch to the right side of the umbilicus, and an inch below it. This tumour was adherent to the integuments, was rather moveable, and there was considerable tenderness on pressure. Notwithstanding the size of the tumour, its tenderness, and its prominent figure, the patient, until my examination, was totally ignorant of its existence. The apparently rapid growth of the tumour, its hardness and irregularity, combined with the bloodless appearance of the patient, and the great and sudden loss of strength experienced, induced me to believe that the disease was of a malignant nature. A dozen leeches were ordered to the part, and a consultation took place in the evening with Dr. Nevinson. Dr. Nevinson was likewise of opinion that the disease was of a malignant kind, but no decision could be formed as to which of the viscera it affected particularly." 232.

Next day Mr. Brodie examined the patient, and agreed in opinion that the disease was fungus hæmatodes. In the course of a few days Sir Astley Cooper investigated the complaint.

" The latter gentleman (Sir A.) was of opinion that the great intestine on the left side adhered to the parietes of the abdomen, that the inner coat had ulcerated, and a tumour was formed whose contents consisted of gas, ill-conditioned matter, and fæces." 233.

On the 23d of March, some fluctuation being perceived, an opening was made with a lancet, and about two ounces of fetid pus were discharged. Some hæmorrhage ensued—the countenance sunk—diarrhœa took place, but was checked. On the 26th there was free discharge from the opening, and the patient seemed to improve; but this amelioration did not last long. The tumour enlarged—and, by the 17th April, it occupied the whole of the umbilical region, being six inches in length and four in breadth, without any pain on pressure. Various means (including iodine) were used to stop the growth of the tumour, but without success. The liquor potassæ, increased to the amount of 25 minims five times in the 24 hours, gave most relief. During three weeks' course of this medicine, a sensible amendment was perceived—the strength increased—the skin assumed a healthier aspect—and the size of the tumour was somewhat diminished. But he gradually fell back, and lingered till the 2d of October, 1827, when he died exhausted, but without pain. During all this protracted illness, digestion was perfectly performed. He lived on animal and farinaceous food—took brandy and water after dinner —and never suffered pain after ingestion—never threw up any of his food. His appetite continued natural till within two days of his death.

*Dissection*. " The body was opened twenty-seven hours after death by Mr. Brodie, assisted by Mr. Cæsar Hawkins. On the external surface of the body several spots of purpura were perceived, and a tumour was easily felt through the parietes of the abdomen, with an opening in its centre, a little above and to the left side of the umbilicus, discharging some dark purulent fluid. The cavity of the abdomen contained about three quarts of water ; on the removal of which, the tumour was found to be formed by the stomach, adhering extensively to the parietes, to which the transverse part of the colon and the omentum were also joined. The stomach was opened on the posterior part, and the cardiac portion and duodenum were found to be quite healthy, the pyloric half alone being the seat of disease. It appeared to consist of a thickening of the coats of this part of the stomach, in some parts above an inch in thickness, with an irregular tumour growing from its whole circumference, of the nature of fungus hæmatodes. The whole interior surface was ulcerated, and several portions of the tumour projected into the cavity of the stomach. The tumour was soft, and highly vascular in the inner part, and gradually became firmer and whiter towards the peritoneal surface, whence several white bands ran in an irregular manner towards the interior of the tumour. The anterior part of the stomach was the thickest, particularly where it adhered to the muscles of the abdomen ; and in it several abscesses were discovered, one of the largest of which was the cavity in which the opening on the surface of the abdomen terminated. The œsophagus near its junction with the stomach contained a small cyst of fluid, resembling an hydatid in appearance, and of the size of a filbert. The liver was rather darker than usual, but otherwise healthy, except that in the left lobe several tubercles were observed of the size of a pea, of a white colour, and of the consistence of soft cartilage. All the other viscera appeared sound." 240.

*Case* 3. John Rae, aged 40, was admitted into St. George's Hospital, under Dr. Hewett, 12th September, 1827. He had received a fall on his back fifteen months previously, which stunned him, but from which he did not appear to sustain any serious injury. Three weeks afterwards, he experienced difficulty in the digestion of his food—that is, in his own words, "his victuals appeared to stop for three or four hours at a spot"—which spot corresponded with the cardia. There was no vomiting. These symptoms, he said, had been removed by some medicine given him. For eight or ten days before coming into the hospital he had had no evacuation from his bowels. By castor oil and injections, some motions were procured, of a dark muddy appearance.

"Towards the right as well as the left hypochondrium and umbilicus, there seems to be some induration of the stomach, and perhaps also of the liver. Pulse 100, regular, soft. Skin natural. Tongue muddy, no yellowness of conjunctiva; he has a peculiarly exsanguine appearance, but has never had any hæmorrhage. That the tumour is of a malignant character, is rendered probable by the expression of countenance, the rapid emaciation, and general progress of symptoms." 241.

Another examination next day left no doubt of the existence of a scirrhus of the stomach, especially of the pyloric orifice. The tongue is clean—appetite good—no inconvenience after eating. Fourteen drops of the solution of the hydriodate of potash (ʒss. to ℥j. of distilled water) were ordered every six hours; and this dose was increased to 20 drops—24 drops, without inconvenience. On the 1st of October, a purging came on, and on the 15th of the same month he died exhausted. In different examinations, while in the hospital, it was observed that a great mass of the tumour varied its situation according to the position of the body.

"*Dissection.* The greater part of the stomach seemed healthy, but at the pylorus a tumour was found as large as a man's fist and nearly globular in shape, occupying the anterior and lower part of the pyloric extremity. A small part projected over and was attached to the duodenum, but most of the tumour formed part of the circumference of the stomach in the situation mentioned, leaving the posterior and upper part of the pylorus free from disease, and not even thickened. The tumour, near its circumference, was hard and white in texture, apparently attached only to the outer part of the coats of the stomach; but in the inner surface of the diseased mass the coat had ulcerated, and a sloughy mass was exposed having a cavity in the centre which communicated with the cavity of the stomach, with irregular projections of a dark brown or blackish colour. The arch of the colon adhered slightly to the tumour, but was unaffected by the disease. A portion of the œsophagus which was cut off with the stomach, and which was about one inch and a half in length, was very much thickened and hardened in its muscular texture, the mucous coat being still healthy, and the cardiac portion of the stomach was also free from disease where it joined the œsophagus." 244.

The liver was much tuberculated, and the branches of the vena portæ were singularly obstructed by a tuberculous encephaloid matter.

There is no doubt but the tumours which are here described are the same as those delineated by Laennec, Andral, and others, under the term "tumeurs encephaloides," but whether they are to be considered as of the same nature with tubercles found in the liver, we are not quite prepared to admit. Dr. S. hazards some speculations on the nature of that disturbance in the animal economy, (circulation, absorption, secretion) which immediately precedes the formation of these diseases.

"It appears to me to present none of the ordinary phenomena of inflammation, nor is its termination in any manner similar to the terminations of that morbid process as far as they are at present understood, as effusion, suppuration, deposition of lymph, or hepatization. It arises often without the unfortunate patient being aware of its commencement, and proceeds without pain, redness, or swelling, or heat of the affected part, these not being observed until its size, or encroachment upon neighbouring parts, produces secondary attacks or alterations in contiguous textures, which rouses the attention of the patient. The exsanguine appearance of the patient, even at a very early period, and the uncommon depression of vital power which he experiences, would lead to the belief of a constitutional cause, either an alteration in the constituents of the blood, from which these diseased products are separated by the ordinary secreting power of vessels, or from a morbid alteration in the secreting powers themselves, or from both of these causes." 248.

Dr. Seymour appends a couple of cases where pain and vomiting, when they are attendants on this disease, "were effectually relieved for a considerable time by the administration of the prussic acid." Some very good plates are added to this paper, illustrative of the morbid structures above described.

---

### XIII.

*On a New Method of preserving Anatomical Preparations.* By JOHN DAVY, M.D. Physician to the Forces, &c.

[Ed. Med.-Chir. Trans. Vol. III.]

WE noticed some short time ago a "new mode of preserving dead subjects," by immersion in molasses—we have now to bring forward quite an opposite mode of preserving from corruption the "frail memorials"—not of man's existence, but of those maladies which have terminated that existence. Dr. Davy's zeal and research in the cause of medical science are very commendable, and we hope they will tend to benefit the profession and society at large. During the last two or three years, our author has been making trials of the method now to be described, and the success has exceeded his most sanguine expectations. Its advantages are triple—it is *cheap*—it is *durable*—and it clearly displays *minute structure.*

"Its great cheapness will be immediately obvious when I mention what it is, and that it is simply the sulphurous acid, which may be prepared in a manner equally economical and easy, by burning sulphur matches over water in any appropriate vessel,* agitating the water when the match ceases to burn, and, when the water is sufficiently impregnated with the acid gas, filtering the solution to render it clear and transparent." 231.

Of the durability of the process Dr. D. has no doubt. He has preparations by him which were made nearly three years ago, and nothing has been done to them all that time—no fluid has been added—no evaporation has taken place—and they are as perfect as when they were first immersed in the acid. They are merely confined in a bottle with a glass stopper, lubricated with a little cerate.

"From the experiments which I have made, the sulphurous acid appears to have nearly the same power in preventing the putrefaction of animal matter, as it has of stopping the fermentation of vegetable juices. I find that serum coagulated by this acid gas, and converted into a kind of jelly, may be kept in water several weeks,

* "The best kind of match for this purpose is that which is used in Italy,—cotton thread dipped in melted sulphur."
